# Styrene Oxide Caused Cell Cycle Arrest and Abolished Myogenic Differentiation of C2C12 Myoblasts

**DOI:** 10.1155/2020/1807126

**Published:** 2020-05-11

**Authors:** Piyaporn Surinlert, Nitchamon Kongthong, Mariam Watthanard, Thannicha Sae-lao, Piyawat Sookbangnop, Chumpol Pholpramool, Chittipong Tipbunjong

**Affiliations:** ^1^Chulabhorn International College of Medicine, Thammasat University, Pathum-Thani 12120, Thailand; ^2^Hatyaiwittayalai School, Songkhla 90112, Thailand; ^3^Faculty of Medicine, Siam University, Bangkok 10160, Thailand; ^4^Department of Biology, Faculty of Science, Prince of Songkla University, Songkhla 90112, Thailand; ^5^Department of Physiology, Faculty of Science, Mahidol University, Bangkok 10400, Thailand; ^6^Department of Anatomy, Faculty of Science, Prince of Songkla University, Songkhla 90112, Thailand; ^7^Gut Biology and Microbiota Research Unit, Prince of Songkla University, Songkhla 90112, Thailand

## Abstract

Contaminations of chemicals in foods and drinks are raising public concerns. Among these, styrene, a monomer for plastic production, receives increasing interest due to its ability to leach from the packaging and contaminate in foods and drinks causing many health problems. The present study was designed to investigate the effects of styrene monomer (STR) and its metabolite styrene oxide (STO) on C2C12 myoblast proliferation and differentiation. Based on an MTT assay, both STR and STO showed no cytotoxic effect at 10–100 *μ*M. However, at 50–100 *μ*M STO, but not STR, significantly inhibited cell proliferation. The STO-treated cells were accumulated in S-phase of cell cycles as revealed by flow cytometry. The antioxidant enzyme (catalase and superoxide dismutase) activities and the gene expressing these enzymes of the arrested cells were decreased and ultimately led to nuclear condensation and expression of apoptotic markers such as cleaved caspase-3 and-9, but not cleaved caspase-8. In addition, STO significantly suppressed myogenic differentiation by decreasing both the number and size of differentiated myotubes. Biochemical analysis showed attenuations of total protein synthesis and myosin heavy chain (MHC) protein expression. In conclusion, a metabolite of styrene, STO, leached from plastic packaging of foods and beverages suppressed both myoblast proliferation and differentiation, which would affect skeletal muscle development and regeneration.

## 1. Introduction

At present, the public concerns about health are increasing. Many people are trying to avoid exposure to harmful chemicals in the products used in daily life. However, they usually overlook contaminations from the packaging of these products. Presently, the most widely used materials for packaging are plastics due to their relatively low costs, convenience, and durability. The chemical monomer of the plastic packaging/container can be leached to the products inside [1]. One of the plastic components that find its way into products resulting in product contamination is styrene monomer [1, 2].

Styrene monomer is an organic compound mainly used for polystyrene and styrene copolymers productions. Polystyrene is widely used for the production of plastic and plastic packaging [3]. Styrene monomer has been reported to leach from the packaging to the foods and beverages [1]. The migration rate increased with increases in temperature and exposure time [2, 4]. Khaksar and Ghazi-Khansari [5] studied the migration of styrene from cups to hot beverages and milk and found that temperature was the most important factor in mass transfer of styrene from the packaging. The derived concentration of styrene monomer in cups to hot beverages was above the EPA (Environmental Protection Agency) recommended level. Among numerous foods, dairy products including milk, cheese, butter, and yoghurt are of particular concern because they are consumed widely. Especially milk, the styrene contamination may cause developmental problems in infants. After entering into the body, the styrene monomer undergoes bioactivation by the cytochrome P450 monooxygenase, resulting the end product, styrene-7,8-oxide (styrene oxide, STO), which is more toxic than styrene [6].

Styrene monomer has been reported to be carcinogenic [7] and can cause various forms of damages to many tissues including the liver and kidneys [8], neuronal cells [9], and cochlear [10, 11]. Although styrene monomer has been reported to markedly affect many tissues, little is known regarding its toxicity in skeletal muscle tissue.

Skeletal muscle is the special tissue which has the capacity to regenerate itself after injury. This capacity belongs to the satellite cells (adult muscle stem cells) located within the basement membrane of the associated muscle fiber. They are in the quiescent stage, with limited gene expression and protein synthesis but are activated by trauma and weight bearing. Following activation, quiescent satellite cells enter the cell cycle, proliferate to produce myoblast progeny, and differentiate to be myotubes needed for muscle repair [12]. Therefore, in this study we investigated the effect of styrene monomer and styrene oxide on C2C12 mouse myoblasts, which is a representative model for muscle development and muscle regeneration.

## 2. Materials and Methods

### 2.1. Chemicals and Reagents

**Figure 1:**
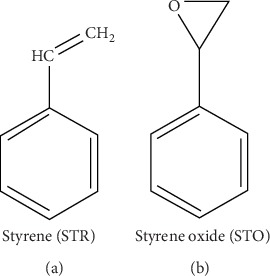
The chemical structure of styrene (a) and styrene oxide (b).

The C2C12 mouse skeletal muscle cell line was purchased from American Type Culture Collection (ATCC, VA, USA). Cell culture reagents and chemicals were from Gibco (Life Technologies, CA, USA). Styrene (STR) and styrene oxide (STO) (Figures 1(a) and 1(b)) and other basic chemical reagents, unless otherwise indicated, were purchased from Sigma-Aldrich (MO, USA). The following antibodies were used in this study: anti-myosin heavy chain (MHC) (Millipore, MA, USA); anti-caspase-9; anti-caspase-8; and anti-caspase-3 (Cell Signaling Technology, MA, USA). Catalase and superoxide dismutase enzyme activity assay kits were obtained from Elabscience (Texas, USA).

### 2.2. Cell Culture and Treatments

C2C12 myoblasts were maintained in a growth medium (GM) composed of DMEM supplemented with 10% fetal bovine serum (FBS) and 1% penicillin/streptomycin at 37°C in a humidified 5% CO_2_ incubator. The C2C12 cell confluence was not allowed to exceed 70%.

To test the cytotoxicity and cell proliferation, C2C12 cells were treated with styrene or styrene oxide in a GM or serum-free medium for the indicated time points.

To induce cell differentiation, C2C12 cells were grown to confluence and were then shifted to a differentiation medium (DM) composed of DMEM supplemented with 2% horse serum (HS) in the absence or presence of styrene or styrene oxide for the indicated time points.

At a designated time, cell morphology was recorded and was then processed for further experiments.

### 2.3. 3-(4,5-Dimethylthiazol-2-yl)-2,5-diphenyltetrazolium Bromide (MTT) Assay

The treated cells were incubated with MTT solution (0.5 mg/ml) diluted in a GM and were then incubated at 37°C for 3 hours. After incubation, the solution was discarded and the formazan crystal was dissolved with a 100 *μ*l solubilizer. The absorbance was determined at 570–630 nm.

### 2.4. Flow Cytometry

The treated cells were collected by trypsinization and fixed with 70% ice-cold methanol. The fixed cells were washed twice with phosphate-buffered saline (PBS) and were then incubated with 0.5 ml RNase A (50 *μ*g/ml) diluted in PBS at 37°C for 30 minutes. After incubation, cells were immediately chilled on ice at least 5 minutes before 100 *μ*l propidium iodide (0.5 mg/ml) was directly added. The reaction was kept in darkness for at least 15 minutes before the cell cycle stage was determined using a BD FACSCanto™ flow cytometer (BD Biosciences, USA) and analyzed with BD FACSDiva version 6.1.1 software.

### 2.5. 2′,7′-Dichlorodihydrofluorescein Diacetate (H2DCFDA) Assay

Reactive oxygen species (ROS) measurement was performed using H2DCFDA. The treated cells were incubated with 5 *μ*M H2DCFDA diluted in a growth medium at 37°C for 30 minutes. After incubation, the solution was discarded and washed several times with PBS. The fluorescence signals were measured with excitation 485 nm and emission 530 nm by using a microplate reader.

### 2.6. Enzyme Activity Assay

The treated cells were collected by trypsinization, and total protein was extracted with radioimmunoprecipitation assay (RIPA) buffer containing 1 : 1000 protease inhibitors cocktail. The extract was then centrifuged at 14,000 rpm, 4°C for 30 minutes. The protein concentration was measured with a bicinchoninic acid assay (BCA) kit. Catalase (CAT) and superoxide dismutase (SOD) were determined with enzyme activity assay kits following the manufacturer's instructions. The enzyme activity was expressed as U/mg protein.

### 2.7. Quantitative Real-Time PCR

After treatment, total RNA was extracted, purified, and converted to cDNA using a cDNA synthesis kit. Quantitative real-time RT-PCR was performed using Luna universal RT-qPCR with BioRad CFX96 Touch Real-Time PCR. Relative gene expression was normalized to GAPDH mRNA, and fold changes compared to control were computed using the 2^−ΔΔCt^ method. Primers used in this study were as follows: SOD1, (forward) 5′-GGAACCATCCACTTCGAGCA-3′ and (reverse) 5′-CCCATGCTGGCCTTCAGTTA-3′, SOD2, (forward) 5′-GCCCAAACCTATCGTGTCCA-3′ and (reverse) 5′-AGGGAACCCCTAAATGCTGCC-3′, SOD3, (forward) 5′-TTCTACGGCTTGCTACTGGC-3′ and (reverse) 5′- GCTAGGTCGAAGCTGGACTC-3′, catalase, (forward) 5′-ACCAAGGTTTGGCCTCACAA -3′ and (reverse) 5′-TCCGGAGTGGGAGAATCCAT-3′, and GAPDH, (forward) 5′-TGCGACTTCAACAGCAACTC-3′ and (reverse) 5′-GCCTCTCTTGCTCAGTGTCC-3′ [13, 14].

### 2.8. Immunofluorescence Staining

The treated cells were fixed with ice-cold methanol, washed, and rehydrated in PBS before blocking the nonspecific binding in 5% normal goat serum for 45 minutes. Primary antibodies were applied and incubated overnight at 4°C. Then, appropriate secondary antibodies conjugated with fluorescence dye were applied and incubated at room temperature for 45 minutes. Hoechst 33342 was stained by mixing with mounting media and was directly applied to the cells. The fluorescence signals were observed under a fluorescence microscope (the Olympus IX73 model). The fusion index of myotubes was determined by counting the number of nuclei in myotubes/total number of nuclei.

### 2.9. Total Protein Extraction and Concentration Determination

Total protein from the treated cells was extracted with RIPA buffer containing protease inhibitor cocktail (1 : 1000). The extract was then centrifuged at 14,000 rpm, 4°C for 30 minutes. The supernatant was collected and subjected to protein concentration determination using a BCA kit.

### 2.10. Western Blot Analysis

An equal amount of protein (20–60 *μ*g) was resolved by 10% SDS-polyacrylamide gel electrophoresis and transferred onto a polyvinylidene difluoride (PVDF) membrane. The membrane was then incubated overnight with the desired primary antibodies such as anti-myosin heavy chain (MHC), anti-caspase-3, anti-caspase-9, anti-caspase-8, and anti-tubulin. The appropriate secondary antibody-conjugated horseradish peroxidase (HRP) was applied, and Western blot signal was detected by film exposure.

### 2.11. Statistical Analysis

All data were derived from at least 3 independent experiments and were expressed as mean ± SEM. Statistical significance was considered when *p* < 0.05 using one-way ANOVA followed by the Tukey multiple comparison test (GraphPad Prism 5.00).

## 3. Results and Discussion

### 3.1. Styrene and Styrene Oxide Are Non-toxic to C2C12 Myoblasts

**Figure 2:**
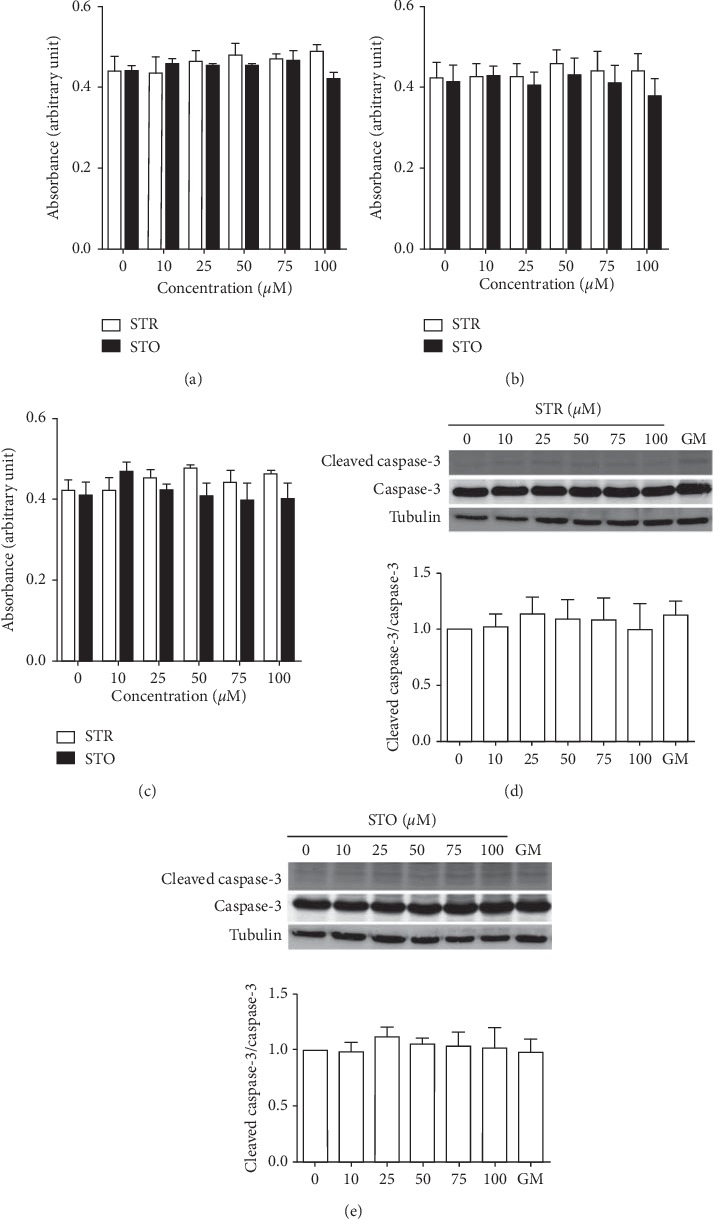
Effects of styrene and styrene oxide on viability of C2C12 myoblasts. The subconfluence C2C12 myoblasts were treated with styrene (open bar) or styrene oxide (the closed bar) at the indicated doses in a serum-free medium for 24 h (a), 48 h (b), and 72 h (c), and then cell viabilities were measured with an MTT assay. The apoptotic marker after treatment with styrene (d) or styrene oxide (e) for 72 h was detected by Western blot analysis with an anti-caspase-3 antibody. GM = serum-containing medium (a growth medium).

**Figure 3:**
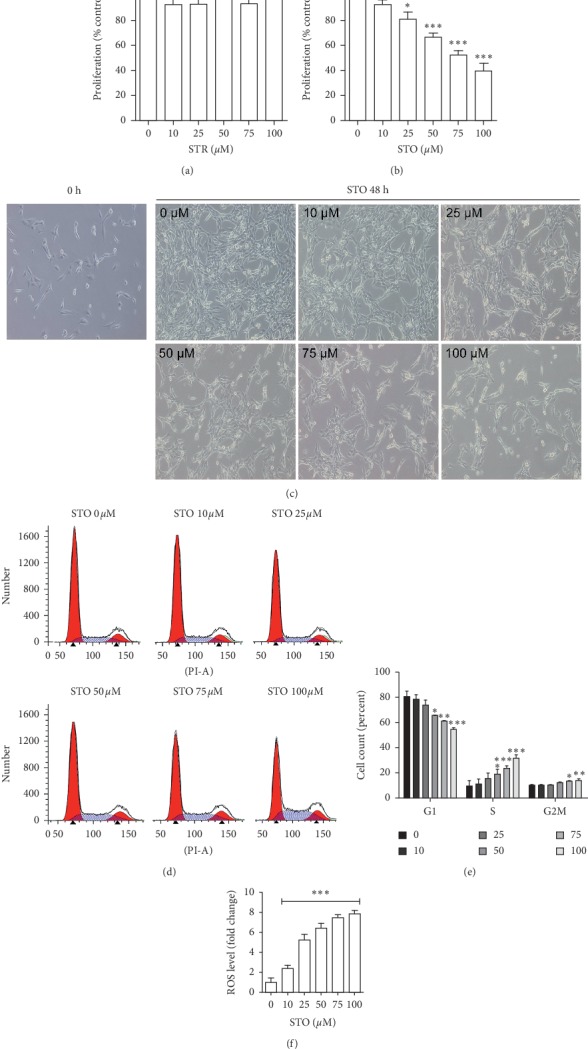
Styrene oxide caused C2C12 myoblast cell cycle arrest in S-phase. The subconfluence C2C12 myoblasts were treated with styrene (a) or styrene oxide (b) at the indicated concentrations in a growth medium for 48 h. After treatment, cell proliferation was detected with the MTT assay and cell morphology was recorded (100x magnification) (c). The cell cycle stage was detected with flow cytometry by staining with propidium iodide (d-e). The level of reactive oxygen species after treatment was measured with the H2DCFDA assay (f). ^*∗*^*p* < 0.05, ^*∗∗*^*p* < 0.01, and ^*∗∗∗*^*p* < 0.001 compared to control.

To study the cytotoxic effects of STR and STO on muscle stem cells, C2C12 myoblasts were treated with various concentrations of STR or STO ranging from 10 *μ*M to 100 *μ*M in a serum-free medium for 24, 48, and 72 h. At the indicated time, cell viability was assessed by an MTT assay. The results showed that treatment of either STR or STO for up to 72 h (Figures 2(a)–2(c)) had virtually no toxic effect on C2C12 myoblasts. Of note, under this condition, both STR and STO failed to induce the expression of cleaved caspase-3, suggesting no induction of apoptosis (Figures 2(d)–2(e)). The lacks of cytotoxic effects of both STR and STO in this study are at variance with the others. Previously, it has been shown that treatment with STR caused an increase in lipid peroxidation (LPO) and reactive oxygen species (ROS) of a rat liver in a dose-dependent manner [15]. Furthermore, *in vitro* study revealed that 50 *μ*M STO induced DNA damages in human peripheral lymphocytes [16, 17]. In addition, an *in vivo* study showed that STR administration for 5 days significantly decreased rat sperm motility and sperm count, and increased sperm abnormality [18]. Such controversy may be due to the dose and exposure time of both STR and STO, as well as cell type being used. The toxic effects of STR and STO were mediated through an increasing level of reactive oxygen species leading to cellular oxidative stress [15, 18–20]. Also in our study, measurements of reactive oxygen species using H2DCFDA showed dose-dependent increases in ROS after treatment with STO (Figure 3(f)).

### 3.2. Styrene Oxide Suppressed C2C12 Myoblast Proliferation by Stimulating Cell Cycle Arrest

To further investigate the effects on cells proliferation, C2C12 myoblasts were treated with STR or STO ranging from 10 *μ*M to 100 *μ*M in a growth medium for 48 h. The MTT results revealed a dose-dependent inhibition of cell proliferation in the STO-treated group, but not in the STR-treated group. STO at the concentration above 25 *μ*M significantly inhibited myoblast proliferation (Figures 3(a)–3(b)). The density of myoblasts in these treated groups was clearly lower than the control group. In particular, at 100 *μ*M, cell numbers in the STO-treated group did not differ from the start (0 h). Moreover, cells morphology of the treated groups developed an elongated cytoplasmic extensions/gripping spicules (Figure 3(c)). Besides, STO markedly affected the cell cycle in a dose-dependent manner as assessed by flow cytometry (Figures 3(d)-3(e)). Treatment with STO exhibited a gradual decrease in cells of G0/G1 phase but an increase in the cell number of S and G2/M phase. Significant changes in the cell cycle stages were observed at the concentration 50 *μ*M and above. These findings suggest that STO had an antiproliferative effect on C2C12 myoblasts by inducing cell cycle arrest in S-phase. The cause of antiproliferative effects of STO in myoblasts may be due to ROS production after STO exposure as previously reported [15]. Indeed, measurements of ROS after treatment with STO significantly increased the level of these free radicals (Figure 3(f)). An induction of ROS by external stimuli has been shown to disrupt cell proliferation by blocking the cell cycle in many cell types including mouse Sertoli cells [21], human pancreatic cancer cells [22], human liver cells [23], and C2C12 myoblasts [24]. In addition, an arrest of myoblast proliferation at the S-phase caused by ROS induction has been reported to occur through the inhibition of the PI3K/Akt/survivin pathway [24].

### 3.3. Styrene Oxide Induced the Onset of Apoptosis

**Figure 4:**
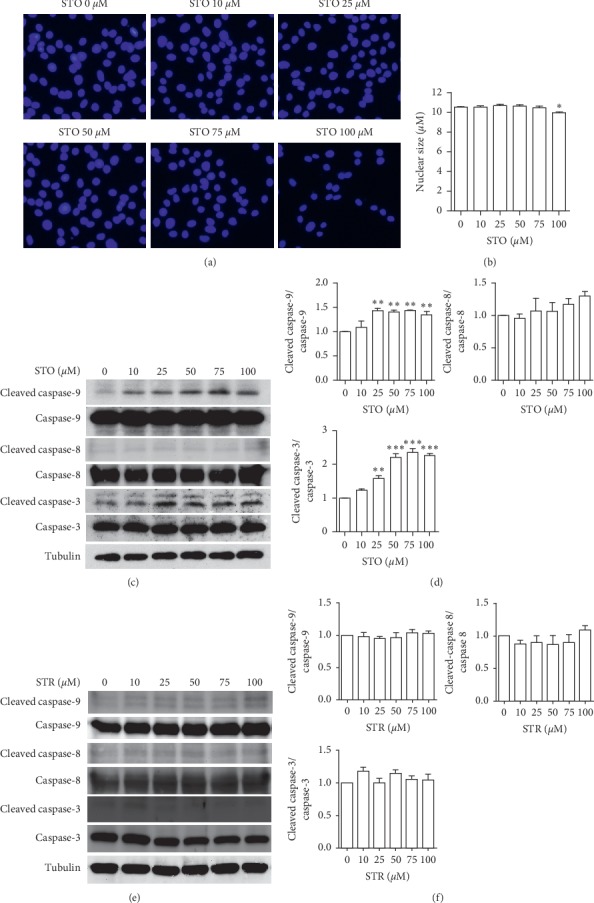
Styrene oxide induced the onset of apoptosis. The subconfluence C2C12 myoblasts were treated with styrene or styrene oxide at the indicated concentrations in a growth medium for 48 h. After treatment, the nucleus was stained with Hoechst 43332 (a) and nuclear size was measured (b). The apoptosis markers in styrene oxide (c-d) or styrene (e-f) treatment were detected by Western blot with anti-caspase-9, anti-caspase-8, and anti-caspase-3 antibodies. ^*∗*^*p* < 0.05, ^*∗∗*^*p* < 0.01, and ^*∗∗∗*^*p* < 0.001 compared to control.

To ascertain whether the accumulated cells in S-phase after STO treatment lead to apoptosis induction, several apoptosis markers were examined. The nuclear staining with Hoechst revealed chromatin condensation after treatment. In particular, the nuclear size was significantly smaller in the 100 *μ*M STO-treated group (9.95 ± 0.12 *μ*M) compared to the nontreated control group (10.55 ± 0.04 *μ*M) (Figures 4(a)–4(b)). We then determined the active form of caspase-9, caspase-3, and caspase-8, which are the mediator proteins of apoptosis. Indeed, treatments with 25–100 *μ*M STO significantly induced the expressions of both cleaved caspase-9 and cleaved caspase-3, but not cleaved caspase-8, compared to control (Figures 4(c)–4(d)). In contrast, treatment with STR showed no effect on the expression of all apoptosis markers being tested such as cleaved caspase-9, cleaved caspase-8, and cleaved caspase-3 (Figures 4(e)–4(f)). The expression of cleaved caspase-9 indicates that STO induced apoptosis partially through the intrinsic pathway. It has been reported that STO caused apoptosis in many cell types including the neuronal PC12 cell line [25], mouse whole lung and Clara cells [19], and cochlear hair cells [26]. The possible mechanism of STO to induce the onset of apoptosis is likely through ROS production. In myoblasts, excess ROS has been reported to mediate apoptosis by inducing mitochondrial dysfunction, endoplasmic reticulum stress, and Akt inactivation pathway [27].

### 3.4. Styrene Oxide Attenuated Antioxidant Enzyme Activity

**Figure 5:**
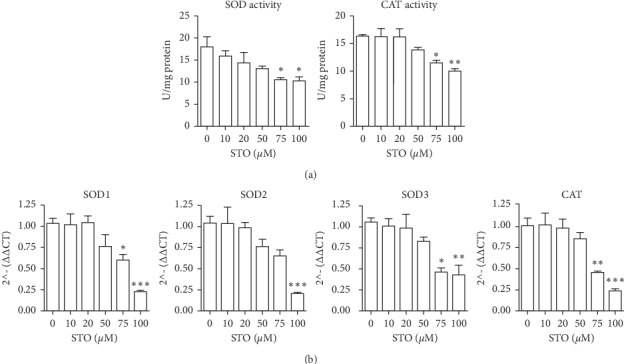
Styrene oxide attenuated antioxidant enzyme activity. The subconfluence C2C12 myoblasts were treated with styrene oxide at the indicated concentrations for 48 h. After treatment, the superoxide dismutase (SOD) and catalase (CAT) enzyme activities were measured (a). The gene expression of antioxidant enzymes, superoxide dismutase 1 (SOD1), superoxide dismutase 2 (SOD2), superoxide dismutase 3 (SOD3), and catalase, were measured by real-time PCR (b). ^*∗*^*p* < 0.05, ^*∗∗*^*p* < 0.01 and ^*∗∗∗*^*p* < 0.001 compared to control.

The levels of ROS and antioxidant enzyme are maintained in a dynamic balance in healthy cells. The onset of apoptosis in C2C12 myoblasts after STO treatment may be caused by disruption of antioxidant enzyme status. A study in rat sperm revealed that STR stimulated ROS production, which, in turn, suppressed antioxidant enzyme expression [18]. Concomitant with our result, treatment with STO showed a trend toward decreases in antioxidant enzyme activity although the effect was not statistically significant at 10–50 *μ*M. However, exposure to STO at 75–100 *μ*M significantly decreased both superoxide dismutase (SOD) and catalase (CAT) enzyme activities in a dose-dependent manner (Figure 5(a)). Moreover, the real-time PCR results showed that treatments with 75–100 *μ*M STO significantly suppressed the expression of antioxidant enzyme genes such as SOD1, SOD2, SOD3, and catalase (Figure 5(b)). Styrene has also been reported to suppress both SOD and CAT expression in sperm cells after systemic administration (500 mg/kg/day) for 5 days [18]. Since these enzymes are responsible for biological system protection from ROS-induced cell death [28], changes in these enzyme activities may prompt the cells leading to apoptosis by removing the defense mechanisms designed to protect cells from oxidative stress.

### 3.5. Styrene Oxide Abolished C2C12 Myoblast Differentiation

**Figure 6:**
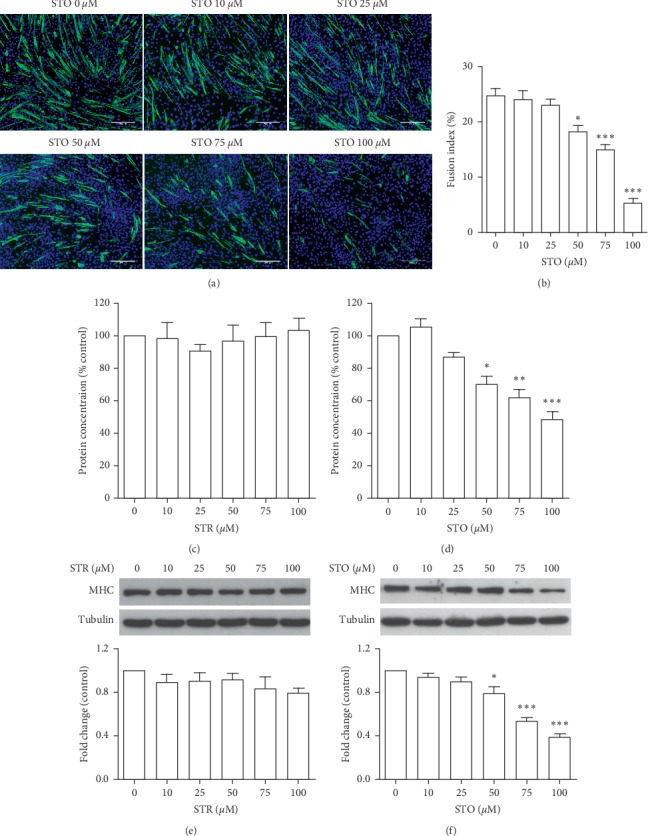
Styrene oxide abolished C2C12 myoblast differentiation. The confluence C2C12 myoblasts were treated with styrene oxide at the indicated concentrations in a differentiation medium for 5 days. After treatment, the differentiated cells were fixed and stained with anti-myosin heavy chain antibody (green) and Hoechst 43332 (blue) (a), and the fusion index was calculated (b). Total protein was extracted, and the concentration was measured (c). The differentiation proteins were detected by Western blot with anti-MHC antibody. ^*∗*^*p* < 0.05, ^*∗∗*^*p* < 0.01, and ^*∗∗∗*^*p* < 0.001 compared to control. Scale bar = 200 *μ*m.

We initially examined the effects of both STR and STO on myoblast differentiation by observing the formation of myotubes for 5 days in differentiation condition. Treatment with STR up to 100 *μ*M did not affect the myotube formation (data are not shown) but STO significantly decreased both the number and size of myotubes in a dose-dependent manner (Figure 6(a)). Exposure to 100 *μ*M STO almost completely abolished myoblast differentiation, only several small myotubes and scattered myocytes were present. The fusion index indicated the failure of myoblast fusion in the presence of STO (Figure 6(b)). We further measured total protein concentration and found the trend of a decrease with increasing STO concentration. The effect was statistically significant at 50–100 *μ*M STO while STR showed no effects on protein synthesis (Figures 6(c)–6(d)). Next, we determined MHC protein expression and found that the protein levels were decreased after treatment with STO, but not after STR (Figures 6(e)–6(f)). Again, the effect was significant at 50–100 *μ*M. Taken together, these results strongly indicate that STO, but not STR, inhibited myoblast differentiation. STO has been reported to affect the differentiation process of rat embryo midbrain and limb bud cells [29]. The possible mechanism was thought to be due to oxidative stress caused by the excess amount of ROS. The oxidative stress conditions have been reported to stimulate the accumulation of S100B, which finally converts the myoblasts into adipocytes via NF-*κ*B/YY1/miR-133 axis and NF-*κ*B/YY1/BMP-7 axis [30]. Moreover, the oxidative stress also inhibits myogenic differentiation via a TLR4-NF-*κ*B-dependent pathway and an autocrine/paracrine TNF-α-induced pathway [31]. Further work is required to assess the mechanism of action of STO on myogenic differentiation.

## 4. Conclusion

This is the first study to evaluate the effects of styrene and its metabolite, styrene oxide, on myoblast cell proliferation and differentiation. Our results revealed the inhibitory effect of styrene oxide on myoblast proliferation by inducing cell cycle arrest and cellular apoptosis. The possible mechanism is likely by suppression of antioxidant enzyme activity. Moreover, we also showed that this chemical inhibited myoblast differentiation. Our findings raise concerns about the potential effect of styrene contamination in foods and drinks on muscle development and regeneration.

## Data Availability

The data used to support the findings of this study are included within the article.
